# Association of Microvascular Function and Endothelial Biomarkers With Clinical Outcome in Dengue: An Observational Study

**DOI:** 10.1093/infdis/jiw220

**Published:** 2016-05-26

**Authors:** Sophie Yacoub, Phung Khanh Lam, Le Hoang Mai Vu, Thi Lien Le, Ngo Thanh Ha, Tran Thi Toan, Nguyen Thu Van, Nguyen Than Ha Quyen, Huynh Thi Le Duyen, Nguyen Van Kinh, Annette Fox, Juthathip Mongkolspaya, Marcel Wolbers, Cameron Paul Simmons, Gavin Robert Screaton, Heiman Wertheim, Bridget Wills

**Affiliations:** 1 Oxford University Clinical Research Unit, Wellcome Trust Major Overseas Programme, Ho Chi Minh City, Hanoi; 2 National Hospital for Tropical Diseases, Hanoi, Vietnam; 3 Department of Medicine, Imperial College London; 4 Nuffield Department of Medicine, University of Oxford, United Kingdom

**Keywords:** Dengue, microcirculation, sidestream dark field imaging, vascular cell adhesion molecule 1, angiopoietin 2

## Abstract

***Background.*** The hallmark of severe dengue is increased microvascular permeability, but alterations in the microcirculation and their evolution over the course of dengue are unknown.

***Methods.*** We conducted a prospective observational study to evaluate the sublingual microcirculation using side-stream dark-field imaging in patients presenting early (<72 hours after fever onset) and patients hospitalized with warning signs or severe dengue in Vietnam. Clinical findings, microvascular function, global hemodynamics assessed with echocardiography, and serological markers of endothelial activation were determined at 4 time points.

***Results.*** A total of 165 patients were enrolled. No difference was found between the microcirculatory parameters comparing dengue with other febrile illnesses. The proportion of perfused vessels (PPV) and the mean flow index (MFI) were lower in patients with dengue with plasma than those without leakage (PPV, 88.1% vs 90.6% [*P* = .01]; MFI, 2.1 vs 2.4 [*P* = .007]), most markedly during the critical phase. PPV and MFI were correlated with the endothelial activation markers vascular cell adhesion molecule 1 (*P* < .001 for both) and angiopoietin 2 (*P* < .001 for both), negatively correlated.

***Conclusions.*** Modest microcirculatory alterations occur in dengue, are associated with plasma leakage, and are correlate with molecules of endothelial activation, angiopoietin 2 and vascular cell adhesion molecule 1.

Dengue is the most prevalent vector-borne viral illness globally, with an estimated annual incidence of approximately 96 million clinically apparent infections [1]. The majority of patients experience a self-limited febrile illness lasting 4–8 days. In a small proportion, however, potentially life-threatening complications develop, including bleeding, organ impairment and, most importantly, plasma leakage that can result in hypovolemic shock [2]. Plasma leakage manifests relatively late in the disease course with hemoconcentration, clinical fluid accumulation, and hemodynamic compromise. However, although the 2009 World Health Organization (WHO) classification highlights a set of warning signs intended to identify patients likely to progress to severe dengue, particularly shock [3], early detection of patients who develop significant vascular leakage remains challenging, and more sensitive methods are needed [4].

The mechanisms underlying the vascular leak syndrome remain poorly understood, but alterations to microvascular function through modulation of the endothelial glyocalyx layer by viral and NS1 protein binding [5–7] and endothelial activation [8] have been implicated. Imbalance of vasoactive mediators, such as T-helper 1 cytokines, angiopoietin (Ang) 1 and 2 [9, 10], vascular endothelial growth factor (VEGF) and its receptors [11], and mast cell products [12], may also play a role.

A number of novel methods to assess microvascular function have become available in recent years, including Sidestream dark field imaging (SDF) imaging, a technique that assesses microcirculatory flow in real-time using videomicroscopy [13]. SDF imaging provides semiquantitative measurement of various parameters that describe vessel density, blood flow, the proportion of perfused vessels, and heterogeneity of flow between vessels. Recent studies using this technique in sepsis and malaria have demonstrated that alterations in the microcirculation are associated with organ failure and worse patient outcomes and have prognostic value independent of global hemodynamics [14, 15]. Mechanisms that may underlie these microcirculatory alterations include reduced perfusion pressure, endothelial dysfunction, reduced red blood cell deformability, and obstruction by sequestered red blood cells in the case of severe malaria [16].

We hypothesized that clinical assessment of microvascular function in dengue could lead to identification of early markers of vascular leakage associated with disease progression. To date, visualization of the microcirculation with SDF imaging has been described in only 2 patients, both with dengue shock syndrome, and the findings showed severe microcirculatory abnormalities with reduced flow and perfusion [17]. However, neither the evolution of microcirculatory alterations during the disease course nor potential associations with clinical outcomes or endothelial activation markers have been evaluated.

Therefore, we set out to investigate changes in the sublingual microcirculation in patients with dengue, and in a comparison group with other febrile illnesses (OFIs), using SDF imaging performed at serial time points throughout the disease course, aiming to explore associations with plasma leakage and patient outcomes. We also evaluated relationships between these microcirculatory parameters and global hemodynamics, as well as with serological markers of endothelial activation.

## METHODS

### Clinical Methods and Patient Recruitment

We performed a STROBE-compliant [18] prospective observational study at the National Hospital for Tropical Diseases (NHTD), Hanoi, Vietnam, between June 2013 and February 2014. Ethical approval was obtained from the Oxford Tropical Research Ethics Committee and the Ethics Review Committee at NHTD, and written informed consent was obtained from all participants or the parents/guardians of children.

Adults and children >5 years of age with a clinical diagnosis of possible dengue were eligible for enrollment into either of 2 study arms. In the outpatient arm, participants presenting within 72 hours of fever onset could be enrolled if no alternative cause for the fever was identified [19]. For the inpatient arm, any patient admitted to NHTD with suspected dengue with warning signs or severe dengue was eligible [3]. All patients were reviewed daily until fully recovered and afebrile, or for up to 6 days after enrollment. Standardized clinical information was recorded daily, including findings of detailed clinical examination and hemodynamic assessment. A complete blood cell count was performed daily, with additional samples obtained for a biochemical profile and dengue diagnostics at enrollment, at defervescence, and at a follow-up visit 10–14 days after illness onset. Any outpatient requiring admission continued to be followed up daily in hospital, with the indication for admission documented, and all management interventions were recorded. Additional investigations, including ultrasonography and/or chest radiology, were performed if clinically indicated.

### Laboratory Investigations

#### Dengue Diagnostics

An NS1 test (Platelia enzyme-linked immunosorbent assay [ELISA]; Bio-Rad) and commercial immunoglobulin (Ig) M and IgG serology assays (Capture ELISA; Panbio) were used to confirm the diagnosis in plasma samples obtained during acute disease and convalescence. In addition, reverse-transcription polymerase chain reaction (RT-PCR) was performed on enrollment samples to identify the viral serotype and measure plasma viremia [20]. Patients were defined as having laboratory-confirmed dengue if the RT-PCR, NS1, or IgM assays were positive at enrollment or if there was IgM seroconversion between paired specimens. A diagnosis of OFI was assigned to participants with no laboratory evidence of acute or recent dengue—that is, if they were negative for RT-PCR, NS1, and IgM/IgG in paired specimens. Patients were considered unclassifiable if they had negative test results at enrollment but no available samples obtained during convalescence.

#### Endothelial Biomarkers

Plasma concentrations of intercellular adhesion molecule 1 (ICAM-1), vascular cell adhesion molecule 1 (VCAM-1), VEGF, E selectin, Ang-1, and Ang-2 were measured at 4 time points: enrollment, 24 hours later, deferevescence or hospital discharge, and follow-up 10–14 days after illness onset. These tests were performed using a magnetic bead–based assay on a Luminex 200 analyzer, according to the manufacturer's specifications (R&D Systems).

### Sublingual Videomicroscopy

SDF imaging was performed using a hand-held videomicroscope with a ×5 objective lens (MicroVision Medical) and disposable lens caps, at the same 4 time points noted above. Image acquisition and subsequent analysis of the videos were performed using AVA 3.2 software, following consensus guidelines described elsewhere [21]. Briefly, video clips were obtained by 3 of the investigators (S. Y., T. L. L., T. T. T.) from 3 different sublingual sites for 60 seconds, with attention to minimize pressure artifacts and reduce. secretions. Videos were analyzed later by S. Y., L. H. M. V., and T. L. L. who were blinded to the time point and the disease category.

The software separates vessels according to their size, with small vessels defined as those <20 µm in diameter, and the total vessel density (TVD) is calculated as the number of vessels identified in the area analyzed. One of the investigators then graded the average flow per quadrant using standardized techniques (0 indicates no flow; 1, intermittent flow; 2, sluggish flow; and 3, normal flow), allowing for calculation of the microvascular mean flow index (MFI). The flow in all vessels was then categorized as normal, intermittent, or absent, and the proportion of perfused vessels (PPV) was calculated. The heterogeneity of flow was determined using the heterogeneity index (HI), (highest − lowest MFI/mean MFI across all sublingual sites.)

Extravasated red blood cells (eRBCs) were graded according to an internally agreed-on classification, because these have not been described using SDF imaging before; the presence of eRBCs was first noted in each quadrant and then scored from 0 to 4, with 0 indicating that no eRBCs were seen, and 4 that eRBCs were noted in all quadrants. The intrasuser and interuser variability of the video analysis were checked at regular intervals and were consistently <10%.

### Portable Echocardiography

For patients enrolled after September 2013, echocardiography was performed at the bedside by one of the investigators (S. Y.), using a SonoSite M-Turbo system with cardiac settings. The echocardiograms were obtained at the same time points as SDF imaging and included 2-dimensional, M-mode, and Doppler studies. Ejection fraction, stroke volume index, and cardiac index were measured according to standardized techniques (see Supplementary Appendix 1).

### Clinical End-Point Definitions

The primary clinical end point was the presence or absence of plasma leakage. A minimum data set was required to fulfill this definition, encompassing all of the following in addition to daily clinical examination: ≥3 hematocrit recordings during acute illness with ≥1 value obtained during the critical period (illness day 4–6); a baseline hematocrit, being the lowest value (in the following order) of the follow-up sample, a sample obtained within 72 hours of fever onset, or, for hospitalized patients only, the discharge sample, provided no parenteral fluid therapy was administered within the preceding 12 hours; and a radiological assessment for vascular leakage within the critical period.

Participants were classified as having no evidence of leakage if the hemoconcentration percentage—(peak − baseline hematocrit/baseline hematocrit) × 100—was <15% and there were no clinical or radiological signs of leakage. Conversely, if the hemoconcentration percentage was ≥15% or any radiological or clinical signs of fluid accumulation were identified, the individual was defined as having significant vascular leakage. We also carried out a sensitivity analysis, omitting the requirement for a radiological examination, assuming that where such a test was not deemed necessary by the treating clinician its results would be likely to be negative. Other outcomes assessed included the presence or absence of mucosal bleeding and overall dengue severity according to the WHO 2009 classification; detailed information on these criteria are included in the Supplementary Appendix.

### Statistical Analysis

Data are presented as frequency (percentage) for categorical variables and median (interquartile range) for continuous parameters. All analyses were defined a priori in a written analysis plan. First, microcirculatory parameters were compared between patients with confirmed dengue and those with OFI in the outpatient arm. Among patients with confirmed dengue in both study arms, associations with plasma leakage and bleeding were then explored. All analyses were based on logistic regression models with dengue diagnosis, plasma leakage, or bleeding as the outcome of interest and the microcirculatory/vascular parameters as covariates. For each covariate, an initial comparison with the outcome of interest used all measurements from the various time points assessed apart from the follow-up values; separate analyses were subsequently performed for each disease phase: early, day of illness 1–3; critical, day of illness 4–6; recovery, day of illness 7–13; and follow-up, after ≥14 days.

Because most patients had multiple microcirculatory measurements performed during the study, potentially with >1 measurement within each disease phase, robust sandwich standard errors based on working independence covariance structure were used throughout the study. Comparisons were adjusted for day of illness, age, sex, and hospitalization, as appropriate.

Associations between microcirculation parameters, serological endothelial biomarkers, and hemodynamic measurements in patients with dengue were assessed by means of partial correlations, controlling for the following potential confounding variables: age, sex, and the day of illness for the measurement. The significance of partial correlations was assessed based on their Fisher transformation and corresponding bootstrap standard errors. The cluster bootstrap, which resamples patients rather than individual parameter values, accounted for multiple measurements per patient. To adjust for multiplicity informally, we used a significance level of .01 for all comparisons. All analyses were performed with the statistical software R (version 3.2.2) and the companion package geepack (version 1.2–0).

## RESULTS

### Patient Data

**Figure 1.**
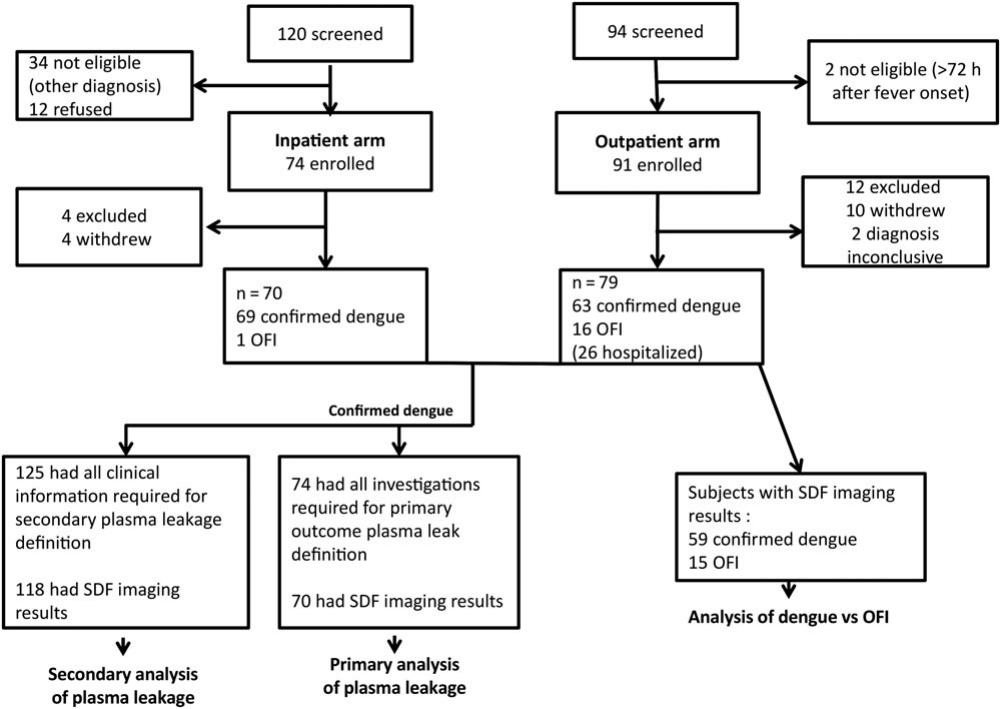
Study flow chart. For primary and secondary analyses of plasma leakage, see Table 3 and Supplementary Table 5, respectively; for analysis of dengue versus other febrile illness (OFI), see Table 2. Abbreviation: SDF, sidestream dark field imaging.

A total of 165 patients were enrolled, 91 in the outpatient arm and 74 in the inpatient arm (Figure 1 and Table 1). After exclusion of 16 patients who withdrew or had an inconclusive diagnosis, 149 patients (79 and 70 in the outpatient and the inpatient arms, respectively) were included in the analysis. Among the outpatients, 63 had laboratory-confirmed dengue and 16 were assigned a diagnosis of OFI; the inpatients included 69 with confirmed dengue and 1 with OFI. Twenty-six outpatients were subsequently hospitalized (24 with confirmed dengue and 2 with OFI), but all participants recovered fully in the end. Considering all study participants, 50% were female and the median age (IQR) was 26 (21–35) years. Inpatients were generally recruited slightly later in the illness course than outpatients, which probably explains the greater derangements observed in the laboratory parameters measured at enrollment. Using the 2009 WHO classification, the 132 patients with confirmed dengue included 57 (43%) with dengue, 68 (52%) with dengue and warning signs, and 7 (5%) with severe dengue (Table 1). Among the 106 patients who were PCR positive, the following serotypes were identified: dengue virus (DENV-1) in 21 (20%), DENV-2 in 18 (17%), DENV-3 in 35 (33%), and DENV-4 in 32 (30%).

**Table 1.** Baseline Characteristics and Clinical Outcomes of Participants

Characteristic	All Patients (n = 149)		Inpatients (n = 70)		Outpatients (n = 79)	
	Patients, No.^a^	Median (IQR) or No. (%)^b^	Patients, No.^a^	Median (IQR) or No. (%)^b^	Patients, No.^a^	Median (IQR) or No. (%)^b^
Baseline						
Age, y	149	26 (21–35)	70	28.5 (21–36)	79	26 (21–33)
Male sex	149	75 (50)	70	36 (51)	79	39 (49)
Day of illness at enrollment	149	3 (3–5)	70	5 (4–6)	79	3 (2–3)
Confirmed dengue	149	132 (89)	70	69 (99)	79	63 (80)
OFI	149	17 (11)	70	1 (1)	79	16 (20)
Platelet count, ×10^9^/L	144	121 (61–161)	67	58 (31–108)	77	151 (126–183)
WBC count, ×10^9^/L	144	3.9 (2.7–5.7)	67	3.4 (2.5–4.6)	77	4.5 (3.0–6.5)
Albumin, g/L	96	44 (40–48)	24	39 (36–45)	72	45 (42–48)
AST, U/L	116	34 (25–57)	40	62 (33–117)	76	32 (23–41)
Clinical outcomes						
Primary end point of plasma leakage^c^	74	38 (51)	47	27 (57)	27	11 (41)
Plasma leakage by clinical definition^d^	125	30 (24)	63	21 (33)	62	9 (15)
Mucosal bleeding	132	59 (45)	69	39 (56)	63	20 (32)
Dengue severity^e^	132		69		63	
Dengue	…	57 (43)	…	19 (28)	…	38 (60)
With warning signs	…	68 (52)	…	45 (65)	…	23 (37)
Severe	…	7 (5)	…	5 (7)	…	2 (3)

Abbreviations: AST, aspartate transaminase; IQR, interquartile range; OFI, other febrile illness; WBC, white blood cell.

^a^ No. of patients with complete data for the respective characteristic.

^b^ No. (%) for categorical variables and median (IQR) for continuous data.

^c^ Primary end-point definition of plasma leakage based on full clinical and radiological information.

^d^ Plasma leakage based on clinical and routine hematological data only.

^e^ Dengue severity according to the World Health Organization 2009 classification.

### Associations Between Microcirculatory Parameters and Dengue Diagnosis

**Figure 2.**
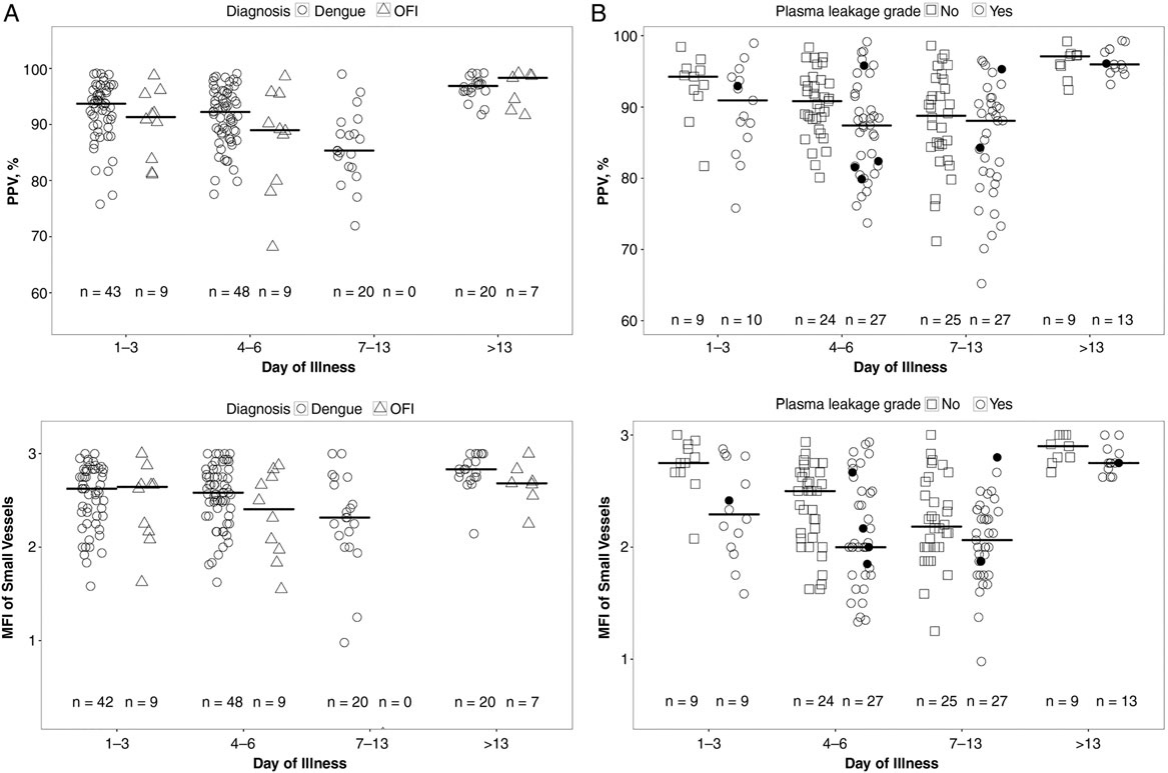
*A*, Scatterplot showing the proportion of perfused vessels (PPV) (*top*), and mean flow index (MFI) (*bottom*), by day of illness in patients with confirmed dengue or other febrile illness (OFI). There were no significant differences between the dengue and OFI groups for either PPV or MFI. *B*, Scatterplot showing the PPV (*top*) and MFI (*bottom*) by day of illness in patients with confirmed dengue with or without plasma leakage (primary end-point definition). There was a significant difference between patients with and those without plasma leakage during acute illness (days 1–13) for both PPV (*P* = .01) and MFI (*P* = .007). Short horizontal lines represent median value of parameter during each illness phase; sample sizes at bottom of each graph, numbers of individuals who contributed to the group (each individual could have >1 measurement during the illness phase); black dots, values from patients who developed dengue shock syndrome.

This analysis was limited to participants enrolled in the outpatient arm of the study. No differences were identified for the microcirculatory variables (small-vessel TVD, PPV, MFI, and HI) between the confirmed dengue (59 participants) and OFI (15 participants) patient groups when considered overall or during any of the individual time periods (Table 2). However, there was a trend toward lower MFI in the patients with OFI than in those with dengue during the critical phase (*P* = .04). Time trends for perfusion and mean flow are shown in Figure 2*A*. Considering all the time points, a higher proportion of patients with dengue had eRBCs seen in ≥1 of the video quadrants (33 of 141; 23%), but this was not significantly different from the proportion in the OFI group (3 of 20; 15%). Collectively, these data indicate similar patterns of microcirculatory disturbance in the dengue and OFI patient groups.

**Table 2.** Small-Vessel Microvascular Variables in Study Participants With Confirmed Dengue Versus OFI, by Disease Phase

Variable by Illness Phase or Day of Illness Group^a^	OFI		Dengue		OR (95% CI)^c^	*P* Value
	Participants, No.; Measurements, No.	Median (IQR)^b^	Participants, No.; Measurements, No.	Median (IQR)^b^		
TVD, mm/mm^3^						
Overall	15; 20	14.2 (12.6–15.5)	59; 143	14.1 (12.6–15.4)	0.92 (.74–1.16)	.49
Day 1–3	9; 10	14.0 (13.1–15.3)	43; 58	13.4 (12.2–15.3)	0.92 (.69–1.22)	.55
Day 4–6	9; 10	14.3 (12.6–15.6)	48; 65	14.1 (12.0–15.3)	0.96 (.68–1.36)	.81
Day 7–13	0; 0	…	20; 20	15.3 (14.3–16.0)	…	…
Day >13	7; 7	14.6 (13.0–16.4)	20; 20	15.8 (14.5–17.4)	1.22 (.70–2.12)	.48
PPV, %						
Overall	15; 20	90.3 (83.2–95.5)	59; 143	92.4 (87.9–95.5)	1.08 (.99–1.17)	.08
Day 1–3	9; 10	91.3 (85.5–94.6)	43; 58	93.7 (90.2–96.1)	1.07 (.94–1.22)	.30
Day 4–6	9; 10	89.0 (82.0–94.2)	48; 65	92.2 (88.5–95.5)	1.11 (.98–1.27)	.10
Day 7–13	0; 0	…	20; 20	85.3 (82.5–88.9)	…	…
Day >13	7; 7	98.3 (93.5–98.7)	20; 20	96.9 (96.0–97.5)	1.12 (.74–1.70)	.60
MFI						
Overall	15; 20	2.6 (2.1–2.7)	59; 142	2.6 (2.3–2.8)	1.27 (.98–1.64)	.07
Day 1–3	9; 10	2.6 (2.2–2.7)	42; 57	2.6 (2.3–2.8)	1.16 (.70–1.92)	.57
Day 4–6	9; 10	2.4 (2.0–2.7)	48; 65	2.6 (2.4–2.8)	1.69 (1.01–2.84)	.04
Day 7–13	0; 0	…	20; 20	2.3 (2.1–2.7)	…	…
Day >13	7; 7	2.7 (2.6–2.8)	20; 20	2.8 (2.8–3.0)	2.45 (.54–11.07)	.25
HI						
Overall	14; 19	0.1 (0.1–0.2)	59; 138	0.2 (0.1–0.3)	1.04 (.80–1.36)	.77
Day 1–3	8; 9	0.2 (0.1–0.3)	41; 56	0.2 (0.1–0.3)	1.00 (.68–1.46)	.98
Day 4–6	9; 10	0.1 (0.1–0.2)	48; 65	0.2 (0.1–0.3)	1.01 (.65–1.58)	.96
Day 7–13	0; 0	…	17; 17	0.2 (0.1–0.4)	…	…
Day >13	6; 6	0.1 (0.0–0.2)	19; 19	0.1 (0.0–0.1)	0.35 (.10–1.22)	.10
eRBC score >0, No. (%)						
Overall	14; 20	3 (15)	59; 141	33 (23)	1.60 (.31–8.28)	.58
Day 1–3	9; 10	2 (20)	42; 56	13 (23)	1.24 (.22–7.18)	.81
Day 4–6	8; 9	1 (11)	48; 65	13 (20)	1.95 (.24–16.06)	.54
Day 7–13	1; 1	0 (0)	20; 20	7 (35)	…	…
Day >13	6; 6	1 (17)	20; 20	0 (0)	…	…

Abbreviations: CI, confidence interval; eRBC, extravasated red blood cell; HI, heterogeneity index; IQR, interquartile range; MFI, mean flow index; OFI, other febrile illness; OR, odds ratio; PPV, proportion of perfused vessels; TVD, total vessel density.

^a^ For each variable, “Overall” corresponds to the overall comparison, which included all values except for those on day of illness >13, adjusted for age, sex, and day of illness. Other rows present comparisons for each day-of-illness category, with adjustment for age and sex. All comparisons were based on generalized estimating equations with independence covariance structure to take into account multiple measurements per patient.

^b^ Data represent median (IQR) except where otherwise indicated (eRBC score).

^c^ The ORs represent the predicted change in the odds of a dengue diagnosis corresponding to increases of 1 mm/mm^3^ in TVD, 1% in PPV, 0.25 unit in MFI (or 1 unit in the total flow MFI from all 4 quadrants), and 0.1 unit in HI and to having a positive eRBC score.

### Associations Between Microcirculatory Parameters and Clinical End Points in Patients With Confirmed Dengue

With respect to plasma leakage, the primary analysis included all patients with confirmed dengue in both arms who had full clinical and radiological assessments for leakage (Table 3). There were no differences identified in total small-vessel density (TVD) between the patients with and those without plasma leakage at any time point. However, the PPV over all time points was lower in patients with plasma leakage (median PPV, 88.1% vs 90.6% for those without leakage; *P* = .01), most markedly during the critical phase (Table 3, Figure 2*B*). The MFI was also lower in the patients with plasma leakage than in those without when considered overall (2.1 vs 2.4, respectively; *P* = .007), and the HI was higher for those with leakage (Table 3). The associations between plasma leakage and PPV, but not between plasma leakage and MFI or HI, were confirmed in the sensitivity analysis using the larger cohort classified as having plasma leakage on the basis of clinical and hematocrit information only (Supplementary Table 5). In terms of prediction, the MFI on day 3 of illness was associated with subsequent plasma leakage (odds ratio per unit increase in MFI, 0.39; 95% confidence interval, .08–.94), although the association did not reach significance at the predefined 1% significance level (*P* = .03) (Supplementary Table 6). The other microcirculatory parameters of TVD, PPV, HI, and eRBC on days 1–3 were not predictive.

**Table 3.** Small-Vessel Microvascular Variables in Patients With Dengue, With or Without Plasma Leakage (Primary End-Point Definition)

Characteristic by Day of Illness^a^	No Leakage		Plasma Leakage		OR (95% CI)^b^	*P* Value
	Participants, No.; Measurements, No.	Median (IQR)	Participants, No.; Measurements, No.	Median (IQR)		
TVD, mm/mm^3^						
Overall	33; 78	14.5 (13.3–5.3)	37; 91	13.3 (11.8–15.0)	0.82 (.68–.98)	.03
Day 1–3	9; 11	14.7 (13.3–16.2)	10; 15	13.4 (11.6–14.7)	0.74 (.51–1.07)	.11
Day 4–6	24; 35	14.7 (13.5–15.4)	27; 39	13.1 (11.6–14.9)	0.84 (.65–1.10)	.21
Day 7–13	25; 32	14.4 (13.4–15.2)	27; 37	13.6 (12.2,-5.1)	0.85 (.67–1.08)	.19
Day >13	9; 9	17.2 (15.7–17.5)	13; 13	14.9 (14.2–15.8)	0.46 (.18–1.19)	.11
PPV, %						
Overall	33; 78	90.6 (85.8–94.0)	37; 91	88.1 (81.6–92.2)	0.94 (.89–.98)	.01
Day 1–3	9; 11	94.3 (92.0–95.3)	10; 15	90.9 (86.7–94.0)	0.93 (.77–1.13)	.49
Day 4–6	24; 35	90.8 (88.3–93.5)	27; 39	87.4 (81.6–91.7)	0.89 (.81–.98)	.02
Day 7–13	25; 32	88.8 (84.5–92.9)	27; 37	88.1 (80.2–90.5)	0.96 (.91–1.02)	.16
Day >13	9; 9	97.1 (95.7–97.3)	13; 13	96.0 (95.3–98.1)	1.53 (.90–2.62)	.12
MFI						
Overall	33; 78	2.4 (2.1–2.7)	37; 90	2.1 (1.9–2.4)	0.75 (.61–.92)	.007
Day 1–3	9; 11	2.8 (2.7–2.9)	9; 14	2.3 (2.0–2.8)	0.24 (.05–1.25)	.09
Day 4–6	24; 35	2.5 (2.1–2.6)	27; 39	2.0 (1.8–2.5)	0.72 (.51–1.02)	.06
Day 7–13	25; 32	2.2 (2.0–2.5)	27; 37	2.1 (1.9–2.3)	0.81 (.58–1.11)	.19
Day >13	9; 9	2.9 (2.8–3.0)	13; 13	2.8 (2.7–2.8)	0.12 (.01–1.33)	.08
HI						
Overall	33; 69	0.2 (0.1–0.2)	37; 81	0.2 (0.1–0.3)	1.33 (1.07–1.66)	.01
Day 1–3	8; 10	0.2 (0.1–0.2)	9; 14	0.3 (0.2–0.4)	2.02 (.95–4.31)	.07
Day 4–6	22; 31	0.2 (0.1–0.3)	27; 35	0.2 (0.1–0.3)	1.02 (.74–1.39)	.91
Day 7–13	21; 28	0.2 (0.1–0.2)	25; 32	0.3 (0.1–0.4)	1.39 (.97–1.99)	.07
Day >13	7; 7	0.1 (0.0–0.1)	10; 10	0.1 (0.0–0.1)	0.18 (.0–11.65)	.42

Abbreviations: CI, confidence interval; HI, heterogeneity index; IQR, interquartile range; MFI, mean flow index; OR, odds ratio; PPV, proportion of perfused vessels; TVD, total vessel density.

^a^ For each variable, “Overall” corresponds to the overall comparison, which included all values except for those on day of illness >14, adjusted for age, sex, hospitalization, and day of illness. Other rows correspond to comparisons for each day-of-illness category, which included all values obtained during that period. All comparisons were based on generalized estimating equations with independence covariance structure to take into account multiple measurements per patient.

^b^ The ORs represent the predicted change in the odds of plasma leakage corresponding to an increase of 1 mm/mm^3^ in TVD, 1% in PPV, 0.25 unit in MFI (or by 1 unit in the total flow MFI from all 4 quadrants), and 0.1 unit in HI.

With respect to bleeding, we found no associations between any of the microcirculatory variables and mucosal bleeding, except a trend for more patients with mucosal bleeding to have eRBCs seen during the early phase (9 of 24 [38%] with vs 8 of 42 [19%] without mucosal bleeding) (Supplementary Table 7). Collectively, these results indicate that although microcirculatory indices of perfusion and flow were worse in patients with plasma leakage than in those without evidence of leakage, the differences were relatively minor.

### Associations Between Microcirculation Parameters, Serological Endothelial Biomarkers, and Global Hemodynamics

**Figure 3.**
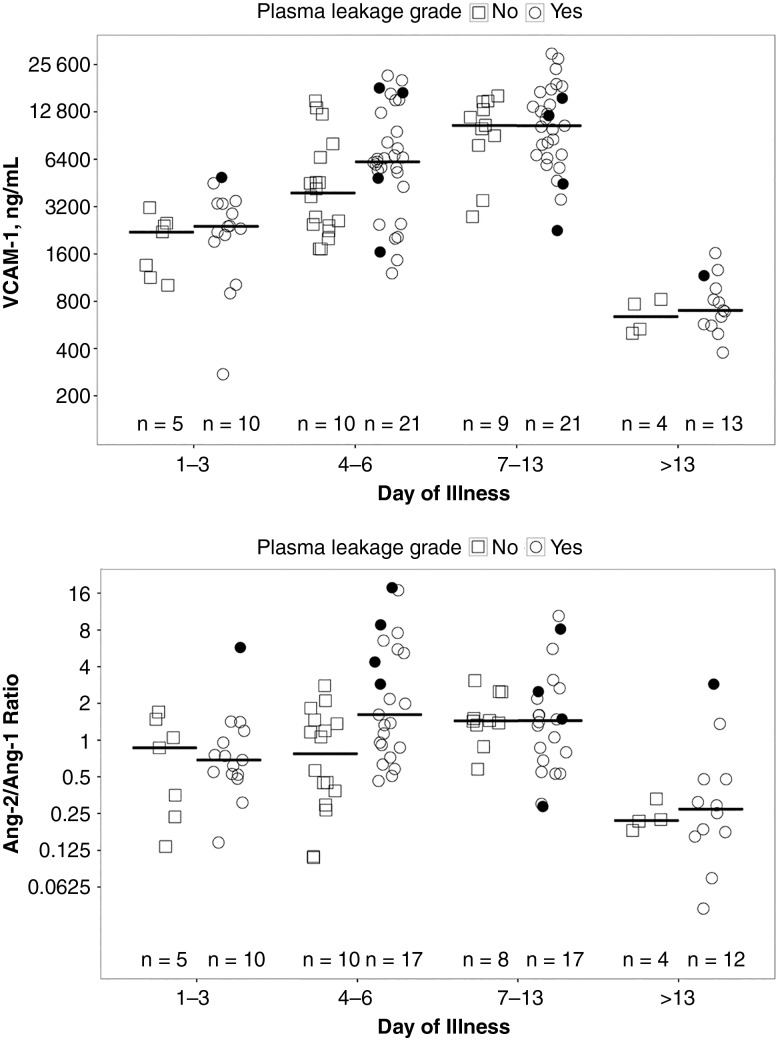
Scatterplot showing vascular cell adhesion molecule 1 (VCAM-1) (*top*) and angiopoietin (Ang) 2/Ang-1 ratio (*bottom*) by day of illness in patients with confirmed dengue with and without plasma leakage (primary end-point definition). Short horizontal lines represent median value of parameter of interest during each illness phase; black dots, values from patients who developed dengue shock syndrome; sample sizes at bottom of each graph, numbers of individuals who contributed to the measurements in that illness phase. There were no significant differences in VCAM-1 levels or Ang-2/Ang-1 ratios between patients with and those without plasma leakage.

Although plasma VCAM-1 levels and Ang-2/Ang-1 ratios were increased during acute dengue illness, there were no statistically significant differences between patients with and patients without plasma leakage (Figure 3), nor were there differences in VEGF and E-selectin levels between these patient groups (data not shown).

Among patients with dengue, PPV and MFI were negatively correlated with molecules associated with endothelial activation, including VCAM-1 (partial correlations, −0.45 for PPV and −0.46 for MFI; both *P* < .001) and Ang-2 (partial correlations, −0.33 and −0.29, respectively; both *P* = .001). No associations were identified between the microcirculatory parameters assessed and the other endothelial biomarkers, including intercellular adhesion molecule 1, E-selectin, VEGF, and Ang-1 (Table 4).

**Table 4.** Partial Correlation Between Small-Vessel Microcirculatory Variables, Endothelial Biomarkers, and Global Hemodynamics in Patients With Dengue

	TVD		PPV		MFI	
Biomarker or Parameter	Rho Value^a^	*P* Value	Rho Value^a^	*P* Value	Rho Value^a^	*P* Value
Serological endothelial biomarkers						
Log_2_ VEGF	–0.254	.06	–0.156	.25	–0.030	.84
Log_2_ ICAM-1	–0.093	.30	–0.136	.18	–0.083	.46
Log_2_ VCAM-1	–0.097	.28	–0.448	<.001	–0.464	<.001
Log_2_ E-selectin	0.165	.15	0.069	.56	–0.002	.98
Log_2_ Ang-1	0.160	.12	0.114	.25	0.123	.21
Log_2_ Ang-2	0.042	.61	−0.332	<.001	–0.290	.001
Global hemodynamic parameters						
Pulse rate	–0.019	.80	–0.005	.94	0.093	.145
MAP	–0.036	.62	0.074	.35	–0.015	.85
Stroke volume index	–0.161	.16	0.096	.44	0.122	.44
Cardiac index	–0.009	.94	0.167	.15	0.256	.02

Abbreviations: Ang-1, angiopoietin 1; Ang-2, angiopoietin 2; ICAM-1, intercellular adhesion molecule 1; MAP, mean arterial pressure; MFI, mean flow index; PPV, proportion of perfused vessels; TVD, total vessel density; VCAM-1, vascular cell adhesion molecule 1; VEGF, vascular endothelial growth factor.

^a^ Partial correlation coefficient between the respective 2 parameters of interest controlling for age, sex, and day of illness of measurement. The significance of partial correlations was assessed based on their Fisher transformation and corresponding bootstrap standard errors.

We found no correlations between the microcirculatory variables and global hemodynamics assessed contemporaneously, such as pulse, mean arterial blood pressure, stroke volume, or cardiac index. Collectively, these results indicate that microcirculatory parameters of flow and perfusion are negatively correlated with endothelial activation markers but not with global hemodynamics.

## DISCUSSION

We have shown that modest microcirculatory disturbances occur in dengue, with a reduction in blood flow and perfusion indices that was most marked in patients with plasma leakage during the critical phase of the disease. MFI and PPV were lower in patients with plasma leakage than in those without when these parameters were assessed overall during the acute illness, and the reduction in PPV was borderline significant during the critical phase (illness day 4–6), but not earlier in the febrile phase. These findings are in agreement with the only other published report assessing the microcirculation in dengue, which showed altered perfusion and flow during the critical phase in 2 severe dengue cases [17]. In addition to flow disturbances, we have shown that eRBCs were visible in a quarter of the patients with dengue assessed, a finding not previously reported in SDF studies. However, unlike flow disturbances the presence of eRBCs was not associated with the clinical outcomes of interest.

Interestingly, these microcirculatory changes do not seem exclusive to dengue, because similar disturbances were seen in the OFI group. Specific diagnoses were not established for the entire OFI group, but they included a mixture of other viral illnesses, such as influenza, measles, and acute hepatitis, as well as some bacterial infections, including scrub typhus. Microcirculatory dysfunction has been demonstrated in numerous studies of severe infections, including sepsis [14], and severe influenza [22], but the current study demonstrates that some degree of microcirculatory change probably also occurs in less severe infections. The majority of microcirculatory studies have been performed in bacterial sepsis, and, similar to our findings, PPV was found to be the most robust parameter associated with outcome [23], although higher HIs were also associated with more severe disease [24].

Our results suggest that microcirculatory parameters assessed in the early febrile phase of dengue have limited prognostic potential, because the flow abnormalities generally occurred later in the disease course and lower MFIs on day 3 were only weakly associated with subsequently developing plasma leakage in the critical phase. This is also in agreement with a study performed in early sepsis, in which microcirculatory abnormalities were not observed in the early phase of low-acuity sepsis [25].

The mechanisms underlying the microcirculatory abnormalities observed in dengue may relate to increased blood viscosity, causing capillary flow to become more sluggish as plasma leakage progresses and hemoconcentration occurs. Endothelial activation may also contribute to the flow disturbances; of the endothelial activation markers we investigated, VCAM-1 and Ang-2 were negatively associated with small-vessel MFI and PPV. A correlation between VCAM-1 and perfusion/flow parameters has been described in pediatric meningococcal sepsis [26]. The causality of the association between VCAM-1 and flow disturbances may be bidirectional, because the expression of VCAM-1 is known to be up-regulated in low blood flow and sheer stress states [27], but VCAM-1–mediated increases in endothelial cell-leukocyte adhesion may also interfere with capillary flow. Ang-2 is known to be up-regulated in vessels with low-flow states [28] and has been implicated in the hemodynamic and microvascular alterations seen in animal models of sepsis [29] and in pulmonary capillary leak in human sepsis [30].

Evidence from human studies and animal models of sepsis also suggests that microcirculation functions independently of systemic hemodynamics, with derangements demonstrated despite normal mean arterial pressure (MAP) and cardiac index [31, 32]. Our data confirm this independence because we found no associations between the microcirculatory parameters and MAP, cardiac output, or stroke volume. This uncoupling of micro- and macrocirculations, together with the observation that microcirculatory parameters correlate more closely with markers of tissue perfusion like lactate, has encouraged critical illness researchers to consider using the microcirculation as a target for goal-directed therapy [33]. Fluid resuscitation has been shown to increase the PPV and flow in early but not late septic shock, independent of MAP, cardiac index, and type of fluid used [34]. Other attempts to improve the microcirculation in sepsis have included use of therapeutics such as activated protein C [35], and corticosteroids [36]. However, larger randomized controlled trials are needed to link these microcirculatory improvements with better organ function and improved patient outcomes in severe infections.

This study had several limitations. First, in the inpatient arm the patients were often enrolled several hours after fluid therapy had commenced, potentially resulting in underestimation of the microcirculatory changes. Second, prediction modeling was limited by the small number of patients experiencing a severe outcome in the outpatient arm. Because patients were generally admitted to the hospital later in the disease course, early assessments (within 72 hours of fever onset) were rarely available for these participants, making comparisons with outpatients difficult. Finally, owing to a certain level of cooperation required by patients undergoing sublingual videomicroscopy, we were unable to study younger children; this age group is at greatest risk for severe plasma leakage, shock, and poor outcomes [37] and may have more marked microcirculatory abnormalities.

In summary, our study has identified moderate abnormalities in microcirculatory flow and perfusion in patients with dengue, with the most severe disturbances seen in those with plasma leakage during the critical phase of the disease. These disturbances were poorly specific, however, because similar changes occurred in patients with OFI. Perfusion indices were correlated negatively with VCAM-1 and Ang-2 levels, suggesting that endothelial activation may underlie the flow disturbances observed in dengue. Although microcirculatory assessment in early dengue is unlikely to be useful for risk prediction, future studies should be considered in established severe dengue to evaluate the utility of including microcirculatory perfusion outcomes alongside other hemodynamic end points in clinical trials assessing novel therapeutic strategies.

## Supplementary Data

Supplementary materials are available at http://jid.oxfordjournals.org. Consisting of data provided by the author to benefit the reader, the posted materials are not copyedited and are the sole responsibility of the author, so questions or comments should be addressed to the author.

Supplementary Data
