# Supplementary material for: Association of Microvascular Function and Endothelial Biomarkers With Clinical Outcome in Dengue: An Observational Study
Source: J Infect Dis. 2016 May 26;214(5):697–706. doi: 10.1093/infdis/jiw220 (PMC4978369; doi:10.1093/infdis/jiw220)
Supplement: Supplementary Data [file supp_214_5_697__index.html]

Association of microvascular function and endothelial biomarkers with clinical outcome in dengue: an observational study — Association of Microvascular Function and Endothelial Biomarkers With Clinical Outcome in Dengue: An Observational Study — Association of Microvascular Function and Endothelial Biomarkers With Clinical Outcome in Dengue: An Observational Study — Supplementary Data 

# Association of Microvascular Function and Endothelial Biomarkers With Clinical Outcome in Dengue: An Observational Study

## Supplementary Data

Supplementary Data

- Supplementary Data - Docx file
- Supplementary Appendix - docx file
