# Supplementary material for: Association of Microvascular Function and Endothelial Biomarkers With Clinical Outcome in Dengue: An Observational Study
Source: J Infect Dis. 2016 May 26;214(5):697–706. doi: 10.1093/infdis/jiw220 (PMC4978369; doi:10.1093/infdis/jiw220)
Supplement: Supplementary Data [file supp_jiw220_jiw220supp.docx]

### Supplementary Table 5. Small vessel microvascular variables in dengue patients with and without the secondary endpoint, clinical plasma leakage

|  | **No leakage** | | |  | **Plasma leakage** | | | **OR** | **(95% CI)** | **p value** |
| --- | --- | --- | --- | --- | --- | --- | --- | --- | --- | --- |
| **Characteristic** | **n** | **N** | **Summary statistic** |  | **n** | **N** | **Summary statistic** |  |  |  |
| **Total vessel density (mm/mm^3^)** | **89** | **203** | **14.2 (12.7-15.3)** |  | **29** | **71** | **12.9 (11.4-14.8)** | **0.82** | **(0.68-1.00)** | **0.046** |
| Day 1-3 | 41 | 53 | 13.9 (12.6-15.5) |  | 9 | 14 | 13.4 (11.7-14.9) | 0.76 | (0.55-1.06) | 0.107 |
| Day 4-6 | 68 | 93 | 14.1 (12.1-15.2) |  | 21 | 29 | 12.4 (11.2-14.5) | 0.90 | (0.71-1.15) | 0.18 |
| Day 7-13 | 42 | 57 | 14.4 (13.3-15.4) |  | 20 | 28 | 13.0 (11.8-14.8) | 0.76 | (0.55-1.03) | 0.080 |
| Day >13 | 24 | 24 | 15.7 (14.5-17.4) |  | 9 | 9 | 14.9 (14.4-15.8) | 0.72 | (0.41-1.26) | 0.248 |
| **Proportion of perfused vessels (%)** | **89** | **203** | **91.4 (87.0-94.6)** |  | **29** | **71** | **88.2 (81.8-92.0)** | **0.94** | **(0.90-0.98)** | **0.003** |
| Day 1-3 | 41 | 53 | 93.7 (89.5-95.7) |  | 9 | 14 | 91.7 (87.8-94.1) | 0.98 | (0.86-1.12) | 0.810 |
| Day 4-6 | 68 | 93 | 91.9 (87.4-95.0) |  | 21 | 29 | 87.2 (81.7-89.6) | 0.90 | (0.85-0.97) | 0.003 |
| Day 7-13 | 42 | 57 | 89.1 (84.6-93.1) |  | 20 | 28 | 86.7 (80.1-90.2) | 0.94 | (0.88-1.00) | 0.039 |
| Day >13 | 24 | 24 | 97.1 (95.9-97.9) |  | 9 | 9 | 95.9 (95.3-96.1) | 0.82 | (0.49-1.39) | 0.465 |
| **Mean Flow Index** | **89** | **203** | **2.5 (2.1-2.7)** |  | **29** | **70** | **2.1 (1.9-2.4)** | **0.81** | **(0.67-0.97)** | **0.026** |
| Day 1-3 | 41 | 53 | 2.6 (2.2-2.8) |  | 8 | 13 | 2.3 (2.1-2.8) | 0.82 | (0.49-1.38) | 0.457 |
| Day 4-6 | 68 | 93 | 2.5 (2.2-2.7) |  | 21 | 29 | 2.0 (1.8-2.5) | 0.72 | (0.54-0.97) | 0.031 |
| Day 7-13 | 42 | 57 | 2.2 (2.0-2.5) |  | 20 | 28 | 2.1 (1.8-2.3) | 0.84 | (0.63-1.13) | 0.252 |
| Day >13 | 24 | 24 | 2.8 (2.7-3.0) |  | 9 | 9 | 2.8 (2.8-2.9) | 0.22 | (0.03-1.39) | 0.107 |
| **Heterogeneity Index** | **89** | **190** | **0.2 (0.1-0.3)** |  | **29** | **62** | **0.2 (0.1-0.3)** | **1.22** | **(0.99-1.50)** | **0.060** |
| Day 1-3 | 40 | 52 | 0.2 (0.1-0.3) |  | 8 | 13 | 0.2 (0.2-0.3) | 1.30 | (0.88-1.92) | 0.187 |
| Day 4-6 | 66 | 89 | 0.2 (0.1-0.3) |  | 21 | 25 | 0.2 (0.1-0.3) | 0.98 | (0.74-1.29) | 0.872 |
| Day 7-13 | 37 | 49 | 0.2 (0.1-0.3) |  | 18 | 24 | 0.3 (0.2-0.4) | 1.42 | (1.04-1.94) | 0.027 |
| Day >13 | 24 | 22 | 0.1 (0.0-0.1) |  | 7 | 7 | 0.0 (0.0-0.1) | 0.19 | (0.0-1.97) | 0.169 |

*Summary statistic is absolute count (%) for categorical variables and median (IQR) for continuous data. n corresponds to number of participants, N corresponds to number of measurements. Day: day of illness. For each variable, the highlighted rows (bolded) correspond to the overall comparison which included all values except for values on day of illness > 14, and was adjusted for age, sex, hospitalization and day of illness. Other rows correspond to comparisons for each day of illness group, which included all values obtained during that time-period. All comparisons were based on generalized estimating equations with independence covariance structure to take into account multiple measurements per patient. The OR describes the predicted change in the odds of plasma leakage corresponding to an increase of 1mm/mm^3^ in TVDs, 1% in PPVs, 0.25 unit in MFIs (or increase by 1 unit in the total flow index from all 4 quadrants), 0.1 unit in HI*

### Supplementary Table 6. Prediction model of microvascular variables on illness day 3 for developing the primary outcome of plasma leakage

| **Covariate** | **No plasma leakage**  **(n = 36)** | |  | **Plasma leakage**  **(n= 37)** | | **OR** | **(95% CI)** | **p value** |
| --- | --- | --- | --- | --- | --- | --- | --- | --- |
|  | **n** |  |  | **n** | **Summary statistics** |  |  |  |
|  |  |  |  |  |  |  |  |  |
| **TVD [mm/mm^3^]** | 9 | 14.3 (13.3, 15.8) |  | 8 | 12.6 (11.4, 13.9) | 0.70 | (0.30-1.19) | 0.209 |
| **PPV [%]** | 9 | 94.4 (92.4, 95.4) |  | 8 | 89.8 (83.0, 93.1) | 0.93 | (0.74-1.12) | 0.467 |
| **MFI** | 9 | 2.8 (2.7, 2.9) |  | 8 | 2.2 (1.9, 2.4) | 0.39 | (0.08- 0.94) | 0.034 |
| **HI** | 8 | 0.2 (0.1, 0.2) |  | 8 | 0.3 (0.2, 0.6) | 1.51 | (0.85-4.18) | 0.179 |
| **eRBC score >0** | 8 | 3 (38%) |  | 8 | 4 (50%) | 1.98 | (0.22-29.48) | 0.552 |

*Summary statistic is absolute count (%) for categorical variables and median (IQR) for continuous data. n corresponds to number of participants. TVD: total small vessel density, PPV: proportion of perfused small vessels, MFI: small vessel mean flow index, HI: heterogeneity index, eRBC: extravasated red blood cells, OR: odds ratio, CI: confidence interval.*

*All comparisons were based on logistic regression with Firth’s correction for separation, and were adjusted for age, sex and hospitalization. The OR corresponds to change in OR for plasma leakage of an increase of 1mm/mm^3^ in TVD, 1% in PPV, 0.25 unit in MFI (or increase by 1 unit in the total flow index from all 4 quadrants), 0.1 unit in HI, having positive eRBC score. This analysis included only dengue patients who had microcirculatory measurements on day 3 of illness and the investigations necessary for the primary plasma leakage outcome.*

### Supplementary Table 7. Small vessel microvascular variables in dengue patients with and without mucosal bleeding

|  |  |  | **No Bleeding** |  |  |  | **Bleeding** | **OR** | **(95% CI)** | **p value** |
| --- | --- | --- | --- | --- | --- | --- | --- | --- | --- | --- |
| **Characteristic** | **n** | **N** | **Summary statistic** |  | **n** | **N** | **Summary statistic** |  |  |  |
| **TVD [mm/mm^3^]** | **70** | **162** | **14.2 (12.3-15.3)** |  | **54** | **123** | **13.5 (12.3-14.9)** | **0.93** | **(0.80-1.07)** | **0.295** |
| Day 1-3 | 32 | 43 | 13.9 (12.3-15.5) |  | 19 | 25 | 13.7 (12.6-15.3) | 0.98 | (0.76-1.25) | 0.868 |
| Day 4-6 | 55 | 78 | 14.2 (12.0-15.2) |  | 36 | 47 | 13.1 (11.9-14.4) | 0.92 | (0.76-1.12) | 0.413 |
| Day 7-13 | 34 | 41 | 14.4 (13.1-15.6) |  | 32 | 51 | 13.9 (12.7-14.9) | 0.89 | (0.71-1.12) | 0.332 |
| Day >13 | 22 | 22 | 15.6 (14.5-17.5) |  | 13 | 13 | 15.0 (14.5-16.5) | 0.89 | (0.59-1.34) | 0.572 |
| **PPV [%]** | **70** | **162** | **91.0 (86.3-94.9)** |  | **54** | **123** | **89.4 (84.5-93.2)** | **0.98** | **(0.94-1.02)** | **0.249** |
| Day 1-3 | 32 | 43 | 93.7 (88.5-96.9) |  | 19 | 25 | 93.1 (89.5-94.5) | 0.98 | (0.88-1.08) | 0.670 |
| Day 4-6 | 55 | 78 | 91.3 (86.9-94.9) |  | 36 | 47 | 88.8 (84.1-92.9) | 0.96 | (0.90-1.02) | 0.209 |
| Day 7-13 | 34 | 41 | 88.5 (84.1-91.8) |  | 32 | 51 | 88.3 (82.6-91.5) | 0.99 | (0.93-1.04) | 0.661 |
| Day >13 | 22 | 22 | 97.1 (96.0-98.0) |  | 13 | 13 | 95.5 (94.8-97.1) | 0.72 | (0.48-1.06) | 0.093 |
| **MFI** | **70** | **162** | **2.5 (2.1-2.7)** |  | **54** | **122** | **2.2 (2.0-2.5)** | **0.87** | **(0.73-1.04)** | **0.120** |
| Day 1-3 | 32 | 43 | 2.6 (2.2-2.8) |  | 18 | 24 | 2.4 (2.2-2.8) | 0.87 | (0.57-1.31) | 0.503 |
| Day 4-6 | 55 | 78 | 2.5 (2.1-2.7) |  | 36 | 47 | 2.3 (2.0-2.5) | 0.87 | (0.68-1.12) | 0.283 |
| Day 7-13 | 34 | 41 | 2.2 (2.0-2.5) |  | 32 | 51 | 2.1 (1.9-2.3) | 0.79 | (0.57-1.09) | 0.144 |
| Day >13 | 22 | 22 | 2.9 (2.8-3.0) |  | 13 | 13 | 2.8 (2.6-2.8) | 0.13 | (0.03-0.49) | 0.003 |
| **HI** | **70** | **148** | **0.2 (0.1-0.3)** |  | **54** | **111** | **0.2 (0.1-0.3)** | **0.96** | **(0.81-1.13)** | **0.614** |
| Day 1-3 | 31 | 42 | 0.2 (0.1-0.3) |  | 17 | 23 | 0.2 (0.1-0.3) | 1.12 | (0.80-1.57) | 0.501 |
| Day 4-6 | 52 | 72 | 0.2 (0.1-0.3) |  | 36 | 44 | 0.2 (0.1-0.3) | 0.92 | (0.74-1.15) | 0.483 |
| Day 7-13 | 29 | 34 | 0.2 (0.1-0.4) |  | 29 | 44 | 0.2 (0.1-0.3) | 0.83 | (0.62-1.10) | 0.195 |
| Day >13 | 17 | 17 | 0.1 (0.0-0.1) |  | 12 | 12 | 0.1 (0.0-0.1) | 5.43 | (0.91-32.53) | 0.064 |
| **eRBC score > 0** | **69** | **157** | **32 (20%)** |  | **53** | **120** | **30 (25%)** | **1.27** | **(0.64-2.54)** | **0.494** |
| Day 1-3 | 32 | 42 | 8 (19%) |  | 18 | 24 | 9 (38%) | 2.56 | (0.82-7.97) | 0.105 |
| Day 4-6 | 54 | 75 | 11 (15%) |  | 36 | 47 | 8 (17%) | 1.52 | (0.50-4.62) | 0.456 |
| Day 7-13 | 33 | 40 | 13 (33%) |  | 31 | 49 | 13 (27%) | 0.83 | (0.33-2.06) | 0.684 |
| Day >13 | 20 | 20 | 0 (0%) |  | 13 | 13 | 0 (0%) | - | - | - |

*Data are presented as absolute count (%) for categorical variables and median (IQR) for continuous data. n corresponds to number of participants, N corresponds to number of measurements. Day: day of illness, TVD: total small vessel density, PPV: proportion of perfused small vessels, MFI: small vessel mean flow index, HI: heterogeneity index, eRBC: extravasated red blood cells, OR: odds ratio, CI: confidence interval.*

*For each variable, highlighted row (bold) corresponds to the overall comparison which included all values except for values on day of illness > 13, and were adjusted for age, sex, hospitalization and day of illness Other rows correspond to comparison for each day of illness group which included all values during that day of illness group. All comparisons were based on generalized estimating equations with independence covariance structure to take into account multiple measurements per patient. The OR corresponds to change in OR for mucosal bleeding of an increase of 1mm/mm^3^ in TVDs, 1% in PPVs, 0.25 unit in MFIs (or increase by 1 unit in the total flow index from all 4 quadrants), 0.1 unit in HI, having positive eRBC score.*
