# Supplementary material for: Association of Microvascular Function and Endothelial Biomarkers With Clinical Outcome in Dengue: An Observational Study
Source: J Infect Dis. 2016 May 26;214(5):697–706. doi: 10.1093/infdis/jiw220 (PMC4978369; doi:10.1093/infdis/jiw220)
Supplement: Supplementary Data [file supp_jiw220_jiw220supp_Appendix.docx]

**Appendix**

**Vascular and cardiac abbreviations**

TVD= Total small vessel density

PPV= Proportion of perfused small vessels

MFI= Small vessel mean flow index

HI = Heterogeneity Index

eRBC= extravasated red blood cells

LVEDD= Left ventricular end diastolic dimension

LVESD = Left Ventricular end systolic dimension

EF= Ejection fraction

VTI= Velocity time Integrals

SV= Stroke volume

SVI= Stroke Volume Index

CO= Cardiac output

CI= Cardiac Index

**Cardiac measurements**

The following measurements were made as per standardized techniques: aortic valve diameter; left ventricular end diastolic (LVEDD) and end systolic (LVESD) dimensions. Ejection fraction was calculated as EF=(LVEDD^3^ – LVESD^3^)/LVEDD^3^%. The aortic velocity from the apical five-chamber view with the sample volume positioned just below the aortic valve cusps, and the Velocity time Integrals (VTIs) were measured at the left ventricular outflow tract (LVOT). Stroke volume was calculated using the equation; VTI x cross sectional area of the aortic valve (CSA), with (CSA= 0.785 x aortic diameter^2^). And cardiac output (CO) =SV x Heart Rate. Cardiac Index (CI) and Stroke Volume Index (SVI) were then calculated by dividing the CO and SV by the body surface area (BSA).

**Dengue disease classification (WHO 2009) [3]**

Patients with confirmed dengue were classified as having dengue, dengue with warning signs, or severe dengue.

If one or more of the following symptoms or signs were present at any time, the patient was classified as having dengue with warning signs:

- Abdominal pain or tenderness on clinical examination, scoring at least 2 out of 3 on pain scale.
- Persistent vomiting- Defined as >2 episodes of vomiting in 24 hours.
- Clinical fluid accumulation- Pleural effusion or ascites detected on clinical examination.
- Mucosal bleed- (including nose, gum, haematuria, abnormal menstrual bleeding).
- Lethargy/ restlessness
- Liver enlargement >2 cm- detected on clinical examination.
- Increase in Haematocrit (HCT) concurrent with rapid decrease in platelet count- Defined as 50% drop in platelet count in 24 hours with any increase in HCT.

If one or more of the following signs were present at any time, the patient was classified as having severe dengue:

- Severe plasma leakage leading to;

1. Shock (Hypotension for age, or pulse pressure of ≤ 20 mmHg with signs of circulatory compromise)

and/or

2) Fluid accumulation with respiratory distress.

- Severe bleeding (bleeding requiring an intervention, e.g. blood transfusion)
- Severe organ involvement

Liver: AST or ALT >=1000

CNS: Impaired consciousness

Heart and other organs (as per clinical assessment)

If none of the above were present at any time the patients was classified as having dengue.
